# Assessment of Second-Generation Diabetes Medication Initiation Among Medicare Enrollees From 2007 to 2015

**DOI:** 10.1001/jamanetworkopen.2020.5411

**Published:** 2020-05-22

**Authors:** Lauren G. Gilstrap, Rachel A. Blair, Haiden A. Huskamp, Katya Zelevinsky, Sharon-Lise Normand

**Affiliations:** 1 Heart and Vascular Center, Dartmouth-Hitchcock Medical Center, Lebanon, New Hampshire; 2 The Dartmouth Institute, Geisel School of Medicine at Dartmouth, Hanover, New Hampshire; 3 Division of Endocrinology, Brigham and Women’s Hospital, Boston, Massachusetts; 4 Department of Health Care Policy, Harvard Medical School, Boston, Massachusetts; 5 Department of Biostatistics, Harvard T.H. Chan School of Public Health, Boston, Massachusetts

## Abstract

**Importance:**

Little is known about how new and expensive drugs diffuse into practice affects health care costs.

**Objective:**

To describe the variation in second-generation diabetes drug use among Medicare enrollees between 2007 and 2015.

**Design, Setting, and Participants:**

This population-based, cross-sectional study included data from 100% of Medicare Parts A, B, and D enrollees who first received diabetes drug therapy from January 1, 2007, to December 31, 2015. Patients with type 1 diabetes were excluded. Data were analyzed beginning in the spring of 2018, and revisions were completed in 2019.

**Exposures:**

For each patient, the initial diabetes drug choice was determined; drugs were classified as first generation (ie, approved before 2000) or second generation (ie, approved after 2000, including dipeptidyl peptidase 4 [DPP-4] inhibitors, glucagon-like peptide-1 [GLP-1] receptor agonists, and sodium-glucose cotransporter-2 [SGLT-2] inhibitors).

**Main Outcomes and Measures:**

The primary outcome was the between-practice variation in use of second-generation diabetes drugs between 2007 and 2015. Practices with use rates of second-generation diabetes drugs more than 1 SD above the mean were considered high prescribing, while those with use rates more than 1 SD below the mean were considered low prescribing.

**Results:**

Among 1 182 233 patients who initiated diabetes drug therapy at 42 977 practices between 2007 and 2015, 1 104 718 (93.4%) were prescribed a first-generation drug (mean [SD] age, 75.4 [6.7] years; 627 134 [56.8%] women) and 77 515 (6.6%) were prescribed a second-generation drug (mean [SD] age, 76.5 [7.2] years; 44 697 [57.7%] women). By December 2015, 22 457 practices (52.2%) had used DPP-4 inhibitors once, compared with 3593 practices (8.4%) that had used a GLP-1 receptor agonist once. Furthermore, 17 452 practices (40.6%) were using DPP-4 inhibitors in 10% of eligible patients, while 1286 practices (3.0%) were using GLP-1 receptor agonists in 10% of eligible patients, and SGLT-2 inhibitors, available after March 2013, were used at least once by 1716 practices (4.0%) and used in 10% of eligible patients by 872 practices (2.0%) by December 2015. According to Poisson random-effect regression models, beneficiaries in high-prescribing practices were more than 3-fold more likely to receive DPP-4 inhibitors (relative risk, 3.55 [95% CI, 3.42-3.68]), 24-fold more likely to receive GLP-1 receptor agonists (relative risk, 24.06 [95% CI, 14.14-40.94]) and 60-fold more likely to receive SGLT-2 inhibitors (relative risk, 60.41 [95% CI, 15.99-228.22]) compared with beneficiaries in low-prescribing practices.

**Conclusions and Relevance:**

These findings suggest that there was substantial between-practice variation in the use of second-generation diabetes drugs between 2007 and 2015, with a concentration of use among a few prescribers and practices responsible for much of the early diffusion.

## Key Points

**Question:**

How have second-generation diabetes drugs diffused into clinical practice among Medicare enrollees?

**Findings:**

In this cross-sectional study including data for 1 182 233 Medicare enrollees who first received diabetes drugs between 2007 and 2015, a time before second-generation diabetes drugs had demonstrated additional cardiovascular benefits and before they were recommended by clinical guidelines, there was substantial variation in their use across practices, with most of the early use concentrated among a few high-prescribing clinicians and practices.

**Meaning:**

This finding suggests that there are potential shortfalls of certain traditional cost containment mechanisms and highlights opportunities to improve the value of early diabetes care.

## Teaser

This cross-sectional study examines patient, prescriber, and practice characteristics associated with first- or second-generation diabetes drug initiation.

## Introduction

In 2015, more than 30 million Americans, almost 10% of the adult population, had diabetes. An additional 1.5 million new cases were being diagnosed annually, the vast majority of which were new cases of type 2 diabetes.^1^ Recent work investigating the factors associated with increased health care spending over the past 17 years found that among all chronic diseases, diabetes had the largest increase in annual spending from 1996 to 2013—an increase of $64 billion, $44 billion (69%) of which was spent on prescription drugs.^2^

Some of this increased spending may be driven by the multiple second-generation diabetes drugs approved by the US Food and Drug Administration (FDA) and released into the market since 2000. Since then, 4 new classes of diabetes drugs have been approved by the FDA for type 2 diabetes. In 2005, the FDA approved amylin analogs and glucagon-like peptide-1 (GLP-1) receptor agonists. The FDA approved dipeptidyl peptidase 4 (DPP-4) inhibitors in 2006 and approved sodium-glucose co-transporter-2 (SGLT-2) inhibitors in 2013.^3^ While each of these drugs offer certain clinical advantages, they are all considerably more expensive than older, first-generation drugs.^4,5,6^ In addition, insulin, a first-generation injectable drug, is a more potent hypoglycemic agent than the GLP-1 receptor agonists or amylin analogs (both injectable medications), and first-generation oral medications (eg, metformin, sulfonylureas) are generally more potent hypoglycemic agents than DPP-4 inhibitors or SGLT-2 inhibitors.^7^

Importantly, between 2007 and 2015, the cardiovascular benefits of second-generation diabetes drugs were suspected but had not yet been proven. It was not until the Empagliflozin Reduces Mortality in Patients With Type 2 Diabetes at High Cardiovascular Risk (EMPA-REG OUTCOME) trial was published in November 2015 that SGLT-2 inhibitors first demonstrated additional cardiovascular benefits.^8^ The cardiovascular benefits of GLP-1 receptor agonists were not proven until June 2016.^9^ Thus, the diffusion of second-generation diabetes drugs as initial therapy between 2007 and 2015 affords a unique opportunity to describe the diffusion and drivers of early drug adoption absent of clear evidence of clinical superiority. To that end, this study aims to use national claims data to quantify the variability in second-generation diabetes drug diffusion between 2007 and 2015 and describe the patient, prescriber, and practice characteristics associated with early diffusion.

## Methods

This project was reviewed and approved by the Harvard Medical School institutional review board. Informed consent was waived, as this was a deidentified, retrospective, claims-based study. This study is reported following the Strengthening the Reporting of Observational Studies in Epidemiology (STROBE) reporting guideline for observational studies.

### Data and Patients

For this cross-sectional study, we used data from 100% of Medicare Parts A, B, and D enrollees to identify beneficiaries 66 years or older with a new diabetes drug fill between 2007 and 2015. Medicare is national health insurance available in the US for people 65 years or older. Medicare A data include claims from inpatient hospitalizations. Medicare B includes data about services not covered by Part A, generally outpatient data. Medicare D data include prescription drug data. In this study, we defined a new diabetes drug fill as 2 or more fills for any diabetes drug at least 30 days but no more than 365 days apart, with no fills of any diabetes drugs in the year prior. To ensure that we had all the prescription information for patients, we required 1 year of continuing Medicare Part D coverage prior to the index diabetes drug fill and 1-year continuing Part D coverage after the fill. We also required 1 year of fee-for-service Medicare coverage prior to the index diabetes drug fill to assess comorbidities. We excluded 62 033 patients with type 1 diabetes based on *International Classification of Diseases, Ninth Revision*^10^ or *Tenth Revision*^11^ codes. A full consort diagram is presented in eFigure 1 in the Supplement.

### Assigning Patients to Prescribers

For each patient, we identified the National Provider Identification (NPI) number of the clinician who prescribed the first diabetes drug. That patient was then attributed to that NPI prescriber.

### Assigning Patients to Practices

We also assigned patients to the practice, identified using tax identification numbers (TINs), responsible for a plurality of their face-to-face office visits using a modified version of the Center for Medicare & Medicaid Services’ 2-step attribution rule.^12,13^ A single practice or TIN can represent a solo practitioner, a small physician group, or a larger health care group. Large organizations will occasionally bill using multiple TINs. Face-to-face visits were identified using evaluation and management *Current Procedural Terminology *(*CPT*) codes 99201 to 99215, 99241 to 99245, G0402, G0438, or G0438, with clinicians who had specialty codes for family practice (08), internal medicine (11), geriatric medicine (38), general practice (01), or endocrinology (46). If patients had the same number of face-to-face visits to more than 1 practice, they were attributed to the practice with the greater sum of allowed spending. Beneficiaries who could not be attributed to a practice or who were attributed to a practice with fewer than 3 patients newly prescribed diabetes therapy in the same year were excluded to ensure adequate intrapractice variability (eFigure 1 in the Supplement).

### Diabetes Drug Classifications

Diabetes drugs were divided into 2 categories based on their date of FDA approval: first-generation drugs were approved before 2000, and second-generation drugs were approved after 2000. All drugs were identified in Part D using National Drug Codes. First-generation diabetes drugs include metformin, sulfonylureas, thiazolidinediones, α-glucosidase inhibitors, meglitinides, and insulin. Second-generation diabetes drugs include amylin analogs (eg, pramlintide), glucagon-like peptide-1 (GLP-1) receptor agonists (eg, exenatide, liraglutide, albiglutide, dulaglutide), dipeptidyl peptidase 4 (DPP-4) inhibitors (eg, sitagliptin, saxagliptin), and sodium-glucose cotransporter 2 (SGLT-2) inhibitors (eg, canagliflozin, dapagliflozin, empagliflozin). Amylin analogues were not considered in our analysis owing to low use rates.

Initiation was defined as the first diabetes drug for which a patient had 2 or more prescriptions, 30 or more days but 365 days or fewer apart. For patients with only 1 fill of multiple diabetes drugs, the first diabetes drug prescribed was considered the initiation drug. Patients who received a combination drug (ie, a single preparation with 2 active ingredients) or combination therapy (ie, 2 drugs prescribed simultaneously) that included a first-generation drug and a second-generation drug, it was considered initiation with the second-generation drug.

### Patient, Clinician, and Practice Characteristics

We determined patient age, sex, race/ethnicity, dual-enrollment status, and disability status directly from the enrollment file. Zip code level estimates for socioeconomic variables, including median household income, percentage of zip code residents 25 years or older who graduated from high school, and percentage of households below the poverty line within the zip code were obtained using the patient’s zip code and 5-year estimates from the 2012 American Community Survey data.^14^ Patient geography was ascertained using the patient’s zip code and the Centers for Disease Control and Prevention’s census regions.^15^ Population density and urbanicity were obtained using the 2010 rural-urban commuting area codes.^16^ Using Chronic Conditions Warehouse flags, we identified patient comorbidities, including acute myocardial infarction, atrial fibrillation, chronic kidney disease, congestive heart failure, hyperlipidemia, hypertension, ischemic heart disease, stroke or transient ischemic attack, and cancer. The community hierarchical condition category score was computed for each beneficiary using Center for Medicare & Medicaid Services’ published algorithm.^17,18^

For each prescriber (ie, NPI), we determined clinician type (ie, endocrinologist, primary care physician, or other type of physician) based on the specialty code used most often for the clinician’s face-to-face visits. We also determined the prescriber’s outpatient panel size (ie, no outpatient panel, <250 patients, 250-500 patients, or >500 patients) using the number of unique face-to-face office visits (identified using the aforementioned *CPT* evaluation and management codes) in each calendar year for each prescriber. Prescribers without any face-to-face office visits in the outpatient or carrier file (ie, clinicians with no outpatient panel) were considered a separate category because they comprised 6.7% of diabetes drug prescribers in 2007 and 10.8% in 2015. Based on a local random sample, these prescribers with no outpatient panel were a heterogeneous group of hospitalists, hospital-based advanced practice clinicians (eg, nurse practitioners or physician assistants), and residents or fellows who bill for inpatient but not outpatient services. Finally, we determined the proportion of patients in a prescriber’s panel with diabetes (<25 patients, 25-50 patients, >50 patients) using Chronic Conditions Warehouse flags for diabetes.

For practices (ie, TINs), we determined the number of all Medicare beneficiaries attributed to the practice in each year, the number of clinicians affiliated with the practice, the ratio of affiliated clinicians to attributed beneficiaries, whether the practice was part of an academic medical center, and whether the practice was hospital-owned. Patients were attributed to the practice where they received most of their face-to-face primary care office visits.^12^ Clinicians were affiliated with the practice through which they submitted most of their face-to-face office visit claims. Whether a practice was part of an academic center was ascertained using the work of Welch and Bindman.^19^ A practice was considered hospital owned if more than 75% of its claims had a place of service code for an outpatient hospital department.^20^

### Outcomes

The primary outcome of this study was between-practice variation in the use of GLP-1 receptor agonists, DPP-4 inhibitors, and SGLT-2 inhibitors between 2007 and 2015. Between-practice variation was defined as the relative risk (RR) that a patient treated at a high-prescribing (defined as ≥1 SD above the mean) practice would receive a second-generation diabetes drug compared with a patient treated at a low-prescribing (≥1 SD below the mean) practice, after adjustment for predetermined patient, prescriber, and practice covariates. Secondary outcomes include the association of prespecified comorbidities with second-generation diabetes drug use rates and the association of clinician and practice characteristics with second-generation diabetes drug use rates.

### Statistical Analysis

We analyzed data collected from January 1, 2007, to December 31, 2015. For continuous variables, we computed means with SDs and medians with interquartile ranges. For categorical variables, we calculated the number and percentage. We then determined the number and percentage of patients started on each diabetes drug in 2007, 2015, and pooled from 2007 to 2015. We determined time to first use of each second-generation diabetes drug for all practices and time to routine use of each second-generation diabetes drug (defined as cumulative 10% use^21^) for each practice. This allowed us to create practice-level diffusion curves that show the percentage of practices that have used each second-generation diabetes drug at least once over time and the percentage of practices that are using each second-generation diabetes drug routinely over time. In our primary analysis, we determined the between-practice variation in use of second-generation diabetes drugs using Poisson random-effects regression models and calculated the RR that a patient treated at a high-prescribing practice would receive a second-generation diabetes drug compared with a patient treated at a low-prescribing practice, after adjustment for patient, prescriber, and practice characteristics that had been determined a priori based on clinical knowledge (eTable 1 in the Supplement). In our secondary analysis, we estimated the association of specific patient comorbidities (ie, chronic kidney disease, ischemic heart disease, and congestive heart failure) and clinician and practice characteristics with the probability of use for each second-generation drug, including a random intercept to account for within-practice correlation. *P* values were 2-tailed and Bonferroni corrected for multiple testing, and significance levels are noted in table footnotes. All statistical analyses were performed using SAS statistical software version 9.4 (SAS Institute). Data were analyzed beginning in the spring of 2018, and revisions were completed in 2019.

## Results

Baseline characteristics of patients initiated on diabetes therapy between 2007 and 2015 are presented in Table 1. This population consisted of 1 182 233 patients cared for by 231 547 prescribers or NPIs in 42 977 practices or TINs. Of these, 1 104 718 patients (93.4%) were prescribed a first-generation diabetes drug and 77 515 patients (6.6%) were prescribed a second-generation diabetes drug as first-line treatment. The proportion of patients dually-eligible for Medicaid was lower among patients who received GLP-1 receptor agonists (540 patients [10.8%]) and SGLT-2 inhibitors (229 patients [11.5%]) than among those who received DPP-4 inhibitors (18 238 patients [25.9%]) or first-generation drugs (239 517 patients [21.7%]). In terms of prescriber and practice characteristics, 5949 patients using second-generation drugs (7.7%) received the prescription from an endocrinologist, 55 290 patients using second-generation drugs (71.3%) received the prescription from a primary care physician, and 8968 patients using second-generation drugs (11.6%) received the prescription from other clinicians. Practices that were affiliated with an academic center prescribed first-generation drugs to a higher proportion of their patients (53 585 patients [4.9%]) than second-generation drugs (GLP-1 receptor agonists: 176 patients [3.5%]; DPP-4 inhibitors: 2215 prescriptions [3.1%]; SGLT-2 inhibitors: 40 patients [2.0%]). Similarly, practices owned by hospitals prescribed first-generation drugs to 83 638 patients (7.7%) , which was a higher proportion than those prescribed second-generation drugs (GLP-1 receptor agonists: 328 patients [6.6%]; DPP-4 inhibitors: 3673 patients [5.2%]; SGLT-2 inhibitors: 82 patients [4.1%]).

**Table 1.** Patient, Clinician, and Practice Characteristics of Patients With Type 2 Diabetes Initiated on Diabetes Therapy Between 2007 and 2015

Characteristic	No. (%)			
	First-generation drugs (n = 1 104 718)^a^	Second-generation drugs (n = 77 451)^b^^,^^c^		
		GLP-1 receptor agonists (n = 4989)	DPP-4 inhibitors (n = 70 471)	SGLT-2 inhibitors (n = 1991)
**Patient level**				
Age, mean (SD), y	75.4 (6.7)	72.1 (4.8)	76.9 (7.2)	73.0 (5.5)
Women	627 169 (56.8)	2958 (59.3)	40 717 (57.8)	987 (49.6)
Race/ethnicity				
White	914 374 (77.3)	52 255 (74.2)	1623 (81.5)	85 6194 (78.0)
Black	98 737 (8.9)	270 (5.4)	6180 (8.8)	132 (6.6)
Hispanic	88 219 (8.0)	247 (5.0)	6364 (9.0)	107 (5.4)
Other	61 632 (5.6)	170 (3.4)	5672 (8.0)	129 (6.5)
Dual Medicare/Medicaid eligibility	239 517 (21.7)	540 (10.8)	18 238 (25.9)	229 (11.5)
Disabled	145 941 (13.2)	782 (15.7)	8394 (11.9)	286 (14.4)
Neighborhood characteristics				
Households below poverty line, % (SD)	11.6 (8.1)	10.3 (7.3)	11.5 (8.3)	9.9 (7.2)
Adults with high school degree, % (SD)	84.9 (9.5)	86.6 (8.6)	84.7 (10.1)	86.3 (8.8)
Household median income, mean (SD), $	53 227 (21 007)	57 401 (23 410)	55 560 (22 974)	58 548 (22 898)
Region				
Northeast	170 877 (15.5)	809 (16.2)	16 348 (23.2)	416 (20.9)
Midwest	278 854 (25.2)	979 (19.6)	13 377 (19.0)	341 (17.1)
South	478 353 (43.3)	2359 (47.3)	29 703 (42.1)	912 (45.8)
West	176 698 (16.0)	842 (16.9)	11 043 (15.7)	322 (16.2)
Population density				
Urban	895 785 (81.1)	4250 (85.2)	60 739 (86.2)	1698 (85.3)
Large rural	107 421 (9.7)	377 (7.6)	5123 (7.3)	154 (7.7)
Small rural	57 559 (5.2)	210 (4.2)	2728 (3.9)	81 (4.1)
Isolated	44 017 (4.0)	152 (3.0)	1881 (2.7)	58 (2.9)
Comorbidities				
Acute myocardial infarction	70 106 (6.4)	185 (3.7)	5345 (7.6)	70 (3.5)
Atrial fibrillation	184 821 (16.7)	773 (15.5)	14 672 (20.8)	311 (15.6)
Chronic kidney disease	286 879 (26.0)	1628 (32.1)	27 339 (38.8)	546 (27.4)
Congestive heart failure	369 439 (33.4)	1683 (33.7)	29 913 (42.4)	527 (26.5)
Hyperlipidemia	959 888 (86.9)	4612 (92.4)	64 726 (91.8)	1813 (91.1)
Hypertension	101 4644 (91.8)	4651 (93.2)	66 898 (94.9)	1851 (93.0)
Ischemic heart disease	626 077 (56.7)	2894 (58.0)	47 276 (67.1)	1066 (53.5)
Stroke or transient ischemic attack	185 777 (16.8)	605 (12.1)	13 676 (19.4)	221 (11.1)
Any cancer	174 606 (15.8)	672 (13.5)	12 255 (17.4)	307 (15.4)
HCC Score^d^	2.2 (1.9)	1.9 (1.5)	2.4 (1.8)	1.7 (1.3)
**Prescriber level**^**e**^				
Position				
Endocrinologist	34 280 (3.1)	811 (16.3)	4949 (7.0)	167 (8.4)
Primary care physician	838 474 (75.9)	3143 (63.0)	50 677 (71.9)	1441 (72.4)
Other physician	131 108 (11.9)	595 (11.9)	8193 (11.6)	180 (9.0)
Panel size, patients				
Mean (SD)	395 (267)	426 (266)	416 (277)	440 (271)
<250	394 682 (35.7)	1625 (32.6)	23 849 (33.8)	623 (31.3)
250-500	422 368 (38.2)	1886 (37.8)	27 308 (38.8)	789 (39.6)
>500	287 732 (26.0)	1478 (29.6)	19 314 (27.4)	579 (29.1)
Patients with diabetes, % (SD)	39.9 (15.5)	48.4 (19.6)	46.5 (18.2)	46.0 (17.8)
<25% patients with diabetes	165 214 (15.0)	615 (12.3)	8784 (12.5)	267 (13.4)
25%-50% patients with diabetes	746 792 (67.6)	2648 (53.1)	39 914 (56.6)	1164 (58.5)
>50% patients with diabetes	192 776 (17.4)	1726 (34.6)	21 773 (30.9)	560 (28.1)
Prescriber has no outpatient panel^f^	70 802 (6.4)	370 (7.4)	5397 (7.7)	178 (9.0)
**Practice level**				
Beneficiaries per practice, mean (SD), No.	6565 (10 222)	6312 (10 289)	5692 (9634)	6636 (10 853)
Clinicians per practice, mean (SD), No.	161 (479)	125 (329)	106 (306)	95 (201)
Beneficiaries per clinician, mean (SD), No.	174 (173)	193 (170)	199 (186)	197 (167)
Part of an academic center	53 587 (5.0)	176 (3.5)	2215 (3.1)	40 (2.0)
Hospital owned	83 638 (7.6)	328 (6.6)	3673 (5.2)	82 (4.1)

Abbreviations: DPP-4, dipeptidyl peptidase 4; GLP-1, glucagon-like peptide-1; HCC, hierarchical condition category; SGLT-2, sodium-glucose co-transporter 2.

^a^ Includes metformin, sulfonylureas, thiazolidinediones, alpha glucosidase inhibitors, meglitinides, and insulin.

^b^ Includes amylin analogs (eg, pramlintide), GLP-1 receptor agonists (eg, exenatide, liraglutide, albiglutide, dulaglutide), DPP-4 inhibitors (eg, sitagliptin, saxagliptin), and SGLT-2 inhibitors (eg, canagliflozin, dapagliflozin, empagliflozin).

^c^ Patients who filled their first 2 prescriptions for a first and second-generation diabetes drug on the same days were classified as initiating the second-generation drug.

^d^ Scores less than 1 are considered relatively healthy, and higher numbers suggest more comorbidities and disease complexity.

^e^ There are 231 547 prescribers in total: 4603 prescribers (2.0%) were endocrinologists, 112 720 prescribers (48.7%) were primary care physicians, and 114 224 (49.3%) were other prescribers.

^f^ Prescriber had no face-to-face office visits in the outpatient or carrier file.

Initiation drug choice evolved significantly between 2007 and 2015 (eTable 2 in the Supplement). Overall, the rate of DPP-4 inhibitor use greatly outpaced that of GLP-1 receptor agonists between 2007 and 2015 (Figure). By 2015, approximately half of practices had used DPP-4 inhibitors once (22 457 practices [52.2%]) compared with 3593 practices (8.4%) having used a GLP-1 receptor agonist once. Similarly, by 2015, 17 452 practices (40.6%) were using DPP-4 inhibitors in 10% of eligible patients, while only 1286 practices (3.0%) were using GLP-1 receptor agonists in 10% of their eligible patients. SGLT-2 inhibitors were not widely available until after March 2013, but their early adoption slope demonstrated more rapid diffusion than GLP-1 receptor agonists, such that by the end of 2015, SGLT-2 inhibitors were used at least once by 1716 practices (4.0%) and used in 10% of eligible patients by 872 practices (2.0%) (Figure). Of note, we considered the diffusion of DPP-4 and metformin combination therapy separately in eFigure 2 in the Supplement. The results are similar but show much lower rates of first and routine DPP-4 inhibitor combination drug use compared with DPP-4 inhibitor–only therapy.

**Figure.**
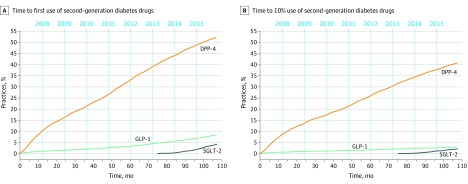
Time to First Use and Time to Routine Use (10% Use) of Second-Generation Diabetes Drugs from 2007 to 2015 The x-axis reflects month of first use starting in January 2007 (month 0) through December 2015 (month 105). Second-generation diabetes drugs included sodium-glucose co-transporter 2 (SGLT-2) inhibitors (ie, canagliflozin, dapagliflozin, and empagliflozin), which were approved March 2013 (ie, month 75); dipeptidyl peptidase 4 (DPP-4) inhibitors (ie, sitagliptin and saxagliptin), which approved October 2006 (metformin with sitagliptin was approved April 2007); and glucagon-like peptide-1 (GLP-1) receptor agonists (ie, exenatide, liraglutide, albiglutide, and dulaglutide), which were approved April 2005. A, Lines reflect the cumulative proportion of practices that have used a second-generation diabetes drug for at least 1 patient in whom they have initiated a diabetes drug. B, Lines reflect the cumulative proportion of practices that have used a second-generation diabetes drug for at least 10% of patients in whom they have initiated a diabetes drug.

In primary analysis, we found significant variation among practices in the diffusion of second-generation diabetes drugs as initial therapy. Patients cared for by high-prescribing practices of DPP-4 inhibitors had an RR of 3.55 (95% CI, 3.42-3.68) to receive a DPP-4 inhibitor compared with patients at low prescribing practices (Table 2). Patients cared for at high-prescribing practices of GLP-1 receptor agonists had an RR of 24.06 (95% CI, 14.14-40.94) to receive a GLP-1 receptor agonist compared with patients cared for at low-prescribing practices. Patients cared for at high-prescribing practices of SGLT-2 inhibitors had an RR of 60.41 (95% CI, 15.99-228.22) to receive an SGLT-2 inhibitor compared with patients cared for at low prescribing practices.

**Table 2.** Between-Practice Variation in Use of Second-Generation Diabetes Drugs Overall and With Specific Comorbidities From 2007 to 2015

Variable	Relative risk (95% CI)		
	SGLT-2 inhibitors	DPP-4 inhibitors	GLP-1 receptor agonists
Overall^a^	60.41 (15.99-228.22)	3.55 (3.42-3.68)	24.06 (14.14-40.94)
Probability of use for patients with specific diseases^b^			
Congestive heart failure	1.08 (0.95-1.23)	NC	NC
*P* value^c^	.21	NC	NC
Chronic kidney disease	NC	1.45 (1.42-1.47)	NC
*P* value^c^	NC	<.001	NC
Ischemic heart disease	NC	NC	1.13 (1.05-1.20)
*P* value^c^	NC	NC	<.001

Abbreviations: DPP-4, dipeptidyl peptidase 4; GLP-1, glucagon-like peptide-1; NC, not calculated; SGLT-2, sodium-glucose co-transporter 2.

^a^ Adjusted for all covariates.

^b^ Adjusted for other demographic characteristics and comorbidities as well as the provider and practice-level covariates.

^c^ *P* values were Bonferroni corrected for 9 tests and 2-sided. *P* < .0056 was considered significant.

To better understand the drivers of this variation, in secondary analyses, we considered predetermined patient, clinician, and practice characteristics. First, we created disease subgroups in which second-generation diabetes drugs had shown early evidence of advantage: chronic kidney disease for DPP-4 inhbitors,^22^ ischemic heart disease for GLP-1 receptor agonists,^23^ and heart failure for SGLT-2 inhibitors.^8^ We then estimated the RR of initiation with each second-generation drug in each disease subgroup (Table 2). Patients with concurrent chronic kidney disease had an RR of 1.45 (95% CI, 1.42-1.47) to receive DPP-4 inhibitor therapy compared with patients without chronic kidney disease (*P* < .001), and patients with concurrent ischemic heart disease had an RR of 1.13 (95% CI, 1.05-1.20) to receive a GLP-1 receptor agonist (*P* < .001). Patients with congestive heart failure were not more likely to receive SGLT-2 inhibitors (RR, 1.08 [95% CI, 0.95-1.23]; *P* = .21), although this may be limited by less than 2 years of data.

Finally, we considered the associations of prescriber and practice characteristics with diffusion variability (Table 3). We found higher rates of second-generation diabetes drug use when the prescriber was an endocrinologist (SGLT-2 inhibitors: RR, 1.88 [95% CI, 1.46-2.42]; *P* < .001; DPP-4 inhibitors: RR, 1.44 [95% CI, 1.37-1.50]; *P* < .001; GLP-1 receptor agonists: RR, 2.35 [95% CI, 2.06-2.68]; *P* < .001) or when the prescribers’ panel had more than 50% of patients with diabetes (SGLT-2 inhibitors: RR, 1.75 [95% CI, 1.34-1.53]; *P* < .001; DPP-4 inhibitors: RR, 1.65 [95% CI, 1.58-1.72]; *P* < .001;GLP-1 receptor agonists: RR, 2.23 [95% CI, 1.91-2.60]; *P* < .001). Prescribers without outpatient panels were more likely than endocrinologists to prescribe second-generation diabetes drugs (SGLT-2 inhibitors: RR, 2.69 [95% CI, 1.68-4.30]; *P* < .001; DPP-4 inhibitors: RR, 1.98 [95% CI, 1.85-2.13]; *P* < .001; GLP-1 receptor agonists: RR, 2.70 [95% CI, 2.03-3.59]; *P* < .001). DPP-4 inhibitors diffused more slowly in practices that were large (RR, 0.64 [95% CI, 0.55-0.75]; *P* < .001), part of an academic center (RR, 0.77 [95% CI, 0.69-0.87]; *P* < .001), or hospital-owned (RR, 0.72 [95% CI, 0.67-0.79]; *P* < .001). GLP-1 diffusion was not associated with any practice characteristics, but similar to DPP-4 inhibitors, SGLT-2 inhibitors also diffused more slowly in hospital-owned practices (RR, 0.48 [95% CI, 0.32-0.75]; *P* < .001).

**Table 3.** Clinician and Practice Characteristics Associated with Use of Second-Generation Diabetes Drug as Initial Therapy From 2007 to 2015

Characteristic	SGLT-2, RR (95% CI)	*P* value^a^	DPP-4, RR (95% CI)	*P* value^a^	GLP-1, RR (95% CI)	*P* value^a^
**Prescriber level**						
Position^b^						
Endocrinologist	1.88 (1.46-2.42)	<.001	1.44 (1.37-1.50)	<.001	2.35 (2.06-2.68)	<.001
Primary care physician	1.32 (1.11-1.55)	.001	0.99 (0.97-1.01)	.43	0.93 (0.84-1.01)	.11
Other	1.03 (0.67-1.57)	.89	0.80 (0.75-0.85)	<.001	0.71 (0.55-0.92)	.009
Panel size, patients						
250-500	1.22 (1.07-1.39)	.002	1.07 (1.05-1.09)	<.001	1.04 (0.96-1.12)	.32
>500	1.25 (1.08-1.45)	.002	1.05 (1.002-1.08)	<.001	1.08 (0.99-1.18)	.08
0^c^	2.69 (1.68-4.30)	<.001	1.98 (1.85-2.13)	<.001	2.70 (2.03-3.59)	<.001
25%-50% with diabetes	1.21 (0.95-1.53)	.11	1.19 (1.15-1.24)	<.001	1.30 (1.13-1.49)	.001
>50% with diabetes	1.75 (1.34-1.53)	<.001	1.65 (1.58-1.72)	<.001	2.23 (1.91-2.60)	<.001
**Practice level**						
>1000 Beneficiaries	1.02 (0.99-1.04)	.16	1.00 (0.99-1.01)	.19	1.02 (1.01-1.04)	.003
>1000 Clinicians	0.54 (0.24-1.25)	.15	0.64 (0.55-0.75)	<.001	0.76 (0.47-1.22)	.25
Part of an academic center	0.48 (0.26-0.89)	.02	0.77 (0.69-0.87)	<.001	0.75 (0.52-1.08)	.12
Hospital owned	0.48 (0.32-0.73)	<.001	0.72 (0.67-0.79)	<.001	0.95 (0.75-1.21)	.68

Abbreviations: DPP-4, dipeptidyl peptidase 4; GLP-1, glucagon-like peptide-1; RR, relative risk; SGLT-2, sodium-glucose co-transporter 2.

^a^ *P* values were Bonferroni corrected for 36 tests and a 2-sided *P* < .0014 was considered significant.

^b^ There are 231 547 prescribers in total: 4603 prescribers (2.0%) were endocrinologists, 112 720 prescribers (48.7%) were primary care physicians, and 114 224 (49.3%) were other prescribers.

^c^ Prescriber had no face-to-face office visits in the outpatient or carrier file.

## Discussion

Between 2007 and 2015, 7% of Medicare patients newly treated for type 2 diabetes were prescribed comparatively expensive second-generation diabetes drugs. By 2015, the most commonly initiated second-generation diabetes drugs were DPP-4 inhibitors, but SGLT-2 inhibitors diffused rapidly after their FDA approval in 2013. Diffusion of GLP-1 was much slower. Overall, we found substantial between-practice variation in the use of these novel therapies, suggesting a concentration of use within a few high prescribers and practices responsible for much of the early diffusion.

To illustrate the difference in cost, during this time, metformin, glipizide, and glyburide (first-generation diabetes drugs) were available as $4 generics while second-generation drugs cost between $300 and $500 per month.^24,25,26^ While claims data may miss some $4 generics fills when patients pay out of pocket, prior data suggest that among Medicare beneficiaries, the rate of use of $4 generic programs is comparatively low (16.3% of beneficiaries) and that even when the $4 generic program is used, a $4 charge for the fill can almost always still be observed in Part D data.^27^ Therefore, the fact that 7% of patients were prescribed markedly more expensive diabetes drugs, either as first or very early second-line therapy, that had not yet demonstrated any cardiovascular morbidity or mortality benefit beyond older first-generation drugs is an important observation.

To explain the difference in diffusion trends among the second-generation diabetes drugs, we hypothesized that specific aspects of the drugs themselves affected early diffusion. For example, the higher rates of use of DPP-4 inhibitors and SGLT-2 inhibitors compared with GLP-1 receptor agonists could potentially be explained by the oral availability of DPP-4 inhibitors and SGLT-2 inhibitors. Normally, the longer drugs are available, the more they diffuse and the less their use becomes concentrated among a small number of clinicians with experience and comfort using them, as illustrated by the RR covariance of DPP-4 inhibitors compared with SGLT-2 inhibitors. In contrast however, the RR covariance of GLP-1 receptor agonists did not decrease over time, suggesting its use remained concentrated among a small group of prescribers and practices over the period of the study. This is likely because GLP-1 receptor agonists are injection drugs, and their use requires more expertise and the ancillary support capacity to teach injection techniques.

Next, we hypothesized that part of the explanation for this higher than anticipated use of expensive second-generation diabetes drugs might have been early signals of benefit in patients with specific comorbidities. For example, as early as 2007, DPP-4 inhibitors were thought to be safer for patients with chronic kidney disease (although most patients require dose reductions), a group of patients for whom metformin is often contraindicated and sulfonylureas have a higher risk of hypoglycemia.^28,29^ Additionally, GLP-1 agonists frequently cause weight loss^30^ and so combined with preliminary data from the Liraglutide Effect and Action in Diabetes: Evaluation of Cardiovascular Outcome Results trial^9^ suggesting a possible decreased rate of cardiovascular death with liraglutide; specifically, GLP-1 agonists may have been particularly attractive for patients with type 2 diabetes and ischemic heart disease. Furthermore, SGLT-2 inhibitors increase glycosuria and thus enhance diuresis,^31^ and, combined with preliminary data from the EMPA-REG OUTCOME study,^8^ it is possible that the clinician anticipated an advantage for SGLT-2 inhibitors in patients with diabetes and congestive heart failure. However, when we examined early diffusion, we found that while there was a higher RR of use in these specific patient subgroups, most of the early use was concentrated among a few high-prescribing clinicians and practices and that these groups were the largest driver of early diffusion.

Consistent with prior work, we found that overall, novel second-generation drugs diffused more rapidly among specialists (ie, endocrinologists) and clinicians who cared for large numbers of patients with diabetes.^32,33^ However, in contrast to prior studies, we also found that a novel group of clinicians—those without outpatient panels—were even more likely than endocrinologists to initiate second-generation diabetes drugs. To better understand who these prescribers were, we identified a random sample of 50 such prescribers from Massachusetts and found that this group was comprised of inpatient-based hospitalists, hospital-based advanced practice clinicians (eg, nurse practitioners), and trainees (ie, residents or fellows). Prior data has suggested that inpatient clinicians may have more exposure to clinical trials^32^ or more experience with complex patients and that these factors may accelerate drug diffusion.^33^ Additionally, we hypothesize that initiating a novel therapy in the inpatient setting may be perceived as lower risk, because laboratory tests can be monitored closely. Moreover, many hospitals now have specialized inpatient services (eg, a diabetes service) and more focused experience to quickly develop a sense of comfort in using novel drugs.

Additionally, this observation raises questions on whether traditional tools that limit expensive drug use, such as prior authorizations, are as effective if the drug is initiated in the inpatient setting or at the time of discharge. In such a case, the burden to prior authorization paperwork may fall on the outpatient clinician. If the patient is doing well and the outpatient clinician is less familiar with the novel drug, the clinician may be more reluctant to change therapies, creating a stronger incentive for outpatient clinicians to scale the regulatory hurdles necessary to continue the drug. If true, this finding highlights the effect that hospital formularies may have on the diffusion of new therapies and suggests the need for alternative regulatory tools to monitor expensive novel therapies initiated in the hospital setting.

### Limitations

Our study has several limitations. First, the results of this study must be interpreted within the context of the population studied. This study examined diffusion trends in older patients (age ≥66 years) with Medicare data. Therefore, the observed trends and patterns may not be applicable to younger populations or those with different insurance coverage. Second, like all claims-based studies, this study is limited by the lack of granular clinical data to further describe the characteristics of patients, clinicians, and practices initiating or being prescribed second-generation diabetes drugs. Third, this study is limited by the incomplete nature of claims data. For example, not all patients had a valid zip code or could be attributed to a valid practice with 3 or more eligible patients, which limited their inclusion in our study. The findings for these populations may be different, although our analysis of baseline characteristics would suggest that they are not (eTable 3 and eTable 4 in the Supplement). Fourth, patients who filled their first 2 prescriptions for a first- and second-generation diabetes drug on the same day were classified as having first received the second-generation drug. While this may slightly increase the rate of second-generation drug use, we found that the rates of initial combination drug use were low. Fifth, this study is observational, so causal conclusions cannot be made.

## Conclusions

The findings of this cross-sectional study suggest that there was significant variation among practices in their use of second-generation diabetes drugs as first-line therapy for diabetes between 2007 and 2015. Before there was any evidence of superior benefit, 7% of Medicare beneficiaries newly prescribed diabetes therapy received an expensive second-generation diabetes drug as first-line therapy. While early signals of benefit and patient comorbidities play a role, clinician and practice patterns played a more significant role. Much of the early use of these drugs was concentrated among a few clinicians and practices that drove most of the early diffusion. This variation in practice patterns highlights a potential shortfall of traditional cost containment mechanisms for prescription drugs and opportunities to improve the value of early diabetes care.

## Notes

Supplement. **eFigure 1.** Consort Diagram **eTable 1.** A Priori Defined Patient, Prescriber, and Practice Characteristics Adjusted for in the Poisson Random Effect Regression Models to Calculate Interpractice Variation **eTable 2.** Initiation Drug Choice in 2007 and 2015 and from 2007 to 2015 **eFigure 2.** Time to First Use and Time to Routine Use of Second-Generation Diabetes Drugs, Including Combination Preparations From 2007 to 2015 **eTable 3.** Comparison of Patients Dropped Owing to Small Practice Size or Inability to Attribute to a Practice vs Those Included in Initiation Cohort **eTable 4.** Comparison of Patients Dropped Owing to Missing Zip Code vs Those Included in Initiation Cohort
