# Supplementary material for: Assessment of Second-Generation Diabetes Medication Initiation Among Medicare Enrollees From 2007 to 2015
Source: JAMA Netw Open. 2020 May 22;3(5):e205411. doi: 10.1001/jamanetworkopen.2020.5411 (PMC7244990; doi:10.1001/jamanetworkopen.2020.5411)
Supplement: Supplement. — eFigure 1. Consort Diagram eTable 1. A Priori Defined Patient, Prescriber, and Practice Characteristics Adjusted for in the Poisson Random Effect Regression Models to Calculate Interpractice Variation eTable 2. Initiation Drug Choice in 2007 and 2015 and from 2007 to 2015 eFigure 2. Time to First Use and Time to Routine Use of Second-Generation Diabetes Drugs, Including Combination Preparations From 2007 to 2015 eTable 3. Comparison of Patients Dropped Owing to Small Practice Size or Inability to Attribute to a Practice vs Those Included in Initiation Cohort eTable 4. Comparison of Patients Dropped Owing to Missing Zip Code vs Those Included in Initiation Cohort [file jamanetwopen-3-e205411-s001.pdf]

Supplementary Online Content

Gilstrap LG, Blair RA, Huskamp HA, Zelevinsky K, Normand S-L. Assessment of second-generation diabetes medication initiation among Medicare enrollees from 2007 to 2015. *JAMA Netw Open*. 2020;3(5):e205411. doi:10.1001/jamanetworkopen.2020.5411

eFigure 1. Consort Diagram
eTable 1. A Priori Defined Patient, Prescriber, and Practice Characteristics Adjusted for in the Poisson Random Effect Regression Models to Calculate Interpractice Variation
eTable 2. Initiation Drug Choice in 2007 and 2015 and from 2007 to 2015
eFigure 2. Time to First Use and Time to Routine Use of Second-Generation Diabetes Drugs, Including Combination Preparations From 2007 to 2015
eTable 3. Comparison of Patients Dropped Owing to Small Practice Size or Inability to Attribute to a Practice vs Those Included in Initiation Cohort
eTable 4. Comparison of Patients Dropped Owing to Missing Zip Code vs Those Included in Initiation Cohort

This supplementary material has been provided by the authors to give readers additional information about their work.

© 2020 Gilstrap LG et al. *JAMA Network Open.*

eFigure 1. Consort diagram

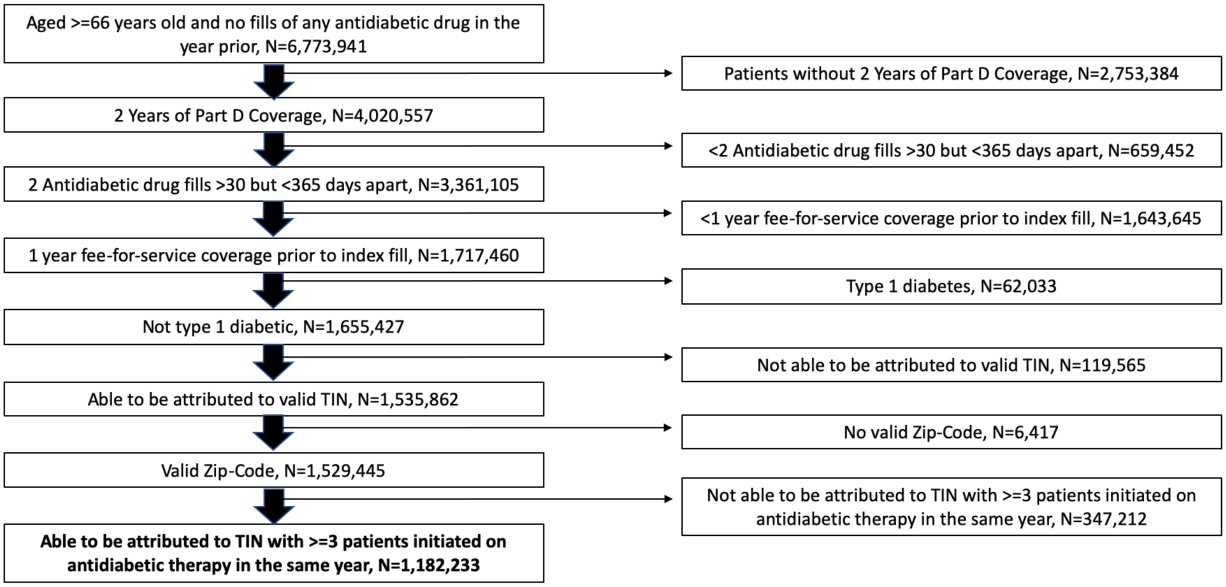

© 2020 Gilstrap LG et al. *JAMA Network Open.*

eTable 1. A Priori Defined Patient, Prescriber, and Practice Characteristics Adjusted for in the Poisson Random Effect Regression Models to Calculate Interpractice Variation

Patient Characteristics
Demographics: Age, sex, race, dual Medicare/Medicaid eligibility, disability, ZCTA-level % below poverty line, ZCTA-level %with high school degree, median income, geographic region, population density
Comorbidities: Acute myocardial infarction, Atrial fibrillation, Chronic kidney disease, Congestive heart failure, Hyperlipidemia, Hypertension, Ischemic heart disease, Stroke/TIA, Cancer, Hierarchical condition category score

Prescriber Characteristics
Prescriber is an Endocrinologist, Prescriber is a primary care physician (PCP), Prescriber is another type of physician (not endocrinologist or PCP), Prescriber's average panel size, % patients in prescriber's panel with diabetes,

Practice Characteristics
Number of beneficiaries attributed to practice, Number of providers affiliated with practice, Number of beneficiaries per provider in practice, Practice is part of an academic center, Practice is hospital owned

© 2020 Gilstrap LG et al. *JAMA Network Open.*

eTable 2. Initiation Drug Choice in 2007 and 2015 and from 2007 to 2015

Initiation Drug	N	%
**2007**		
Initiated on a First-Generation Antidiabetic Drug (n=138516)		
Metformin, n (%)	66867	46.4
* Sulfonylurea, n (%)	39095	27.2
† Thiazolidinediones, n (%)	19703	13.7
‡ Alpha-glucosidase Inhibitors, n (%)	204	0.1
§ Meglitinides, n (%)	1913	1.3
ǀ Insulin, n (%)	10734	7.5
Initiated on a Second-Generation Antidiabetic Drug (n=5468)		
# Amylin Analogue, n (%)	17	<0.1
** GLP-1, n (%)	390	0.3
†† DPP-4, n (%)	5061	3.5
‡‡ SGLT-2, n (%)	-	-
**2015**		
Initiated on a First-Generation Antidiabetic Drug (n=148010)		
Metformin, n (%)	107351	65.88
* Sulfonylurea, n (%)	22786	13.98
† Thiazolidinediones, n (%)	2151	1.32
‡ Alpha-glucosidase Inhibitors, n (%)	209	0.13
§ Meglitinides, n (%)	977	0.6
‖ Insulin, n (%)	14536	8.92
Initiated on a Second-Generation Antidiabetic Drug (n=14929)		
# Amylin Analogue, n (%)	N/A	N/A
** GLP-1, n (%)	1377	0.85
†† DPP-4, n (%)	12181	7.48
‡‡ SGLT-2, n (%)	1371	0.84
**2007-2015 (pooled)**		
Initiated on a First-Generation Diabetes Drug (n=1,104,718)		
Metformin, n (%)	690612	58.42
* Sulfonylurea, n (%)	242126	20.48
† Thiazolidinediones, n (%)	51710	4.37
‡ Alpha-glucosidase Inhibitors, n (%)	1994	0.17
§ Meglitinides, n (%)	10140	0.86
‖ Insulin, n (%)	108136	9.15
Initiated on a Second-Generation Antidiabetic Drug (n=77,515)		
# Amylin Analogue, n (%)	64	<0.1
** GLP-1, n (%)	4989	0.42
†† DPP-4, n (%)	70471	5.96
‡‡ SGLT-2, n (%)	1991	0.17

All combination drugs where one of the active ingredients is metformin are counted as “metformin” except metformin+sitagliptin which is counted as sitagliptin (DPP-4).
* Sulfonylureas include: glipizide, glyburide, glimepiride.
† Thiazolidinediones include: rosiglitazone, pioglitazone.
‡ Alpha-glucosidase inhibitors include: acarbose, miglitol.
§ Meglitinides: repaglinide, nateglinide.
|| Insulins include: Short acting - aspart, glulisine, regular, lispro, human (inhaled))and Long acting - NPH, glargine, degludec, detemir.

© 2020 Gilstrap LG et al. *JAMA Network Open.*

eFigure 2. Time to First Use and Time to Routine Use of Second-Generation Diabetes Drugs, Including Combination Preparations From 2007 to 2015

Time to First Use

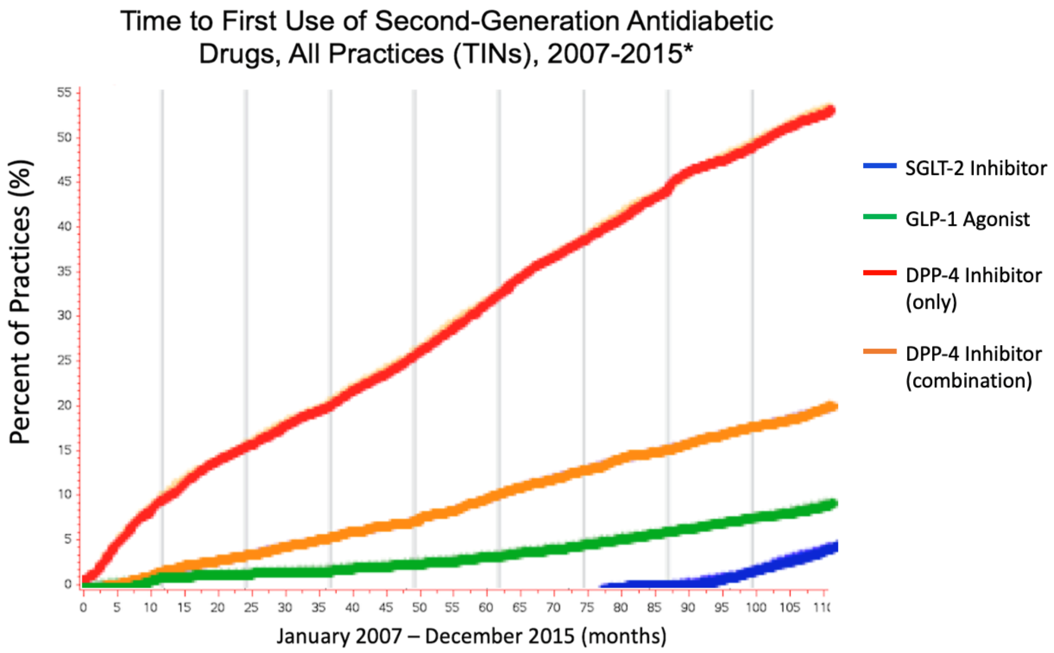

Time to 10% Use

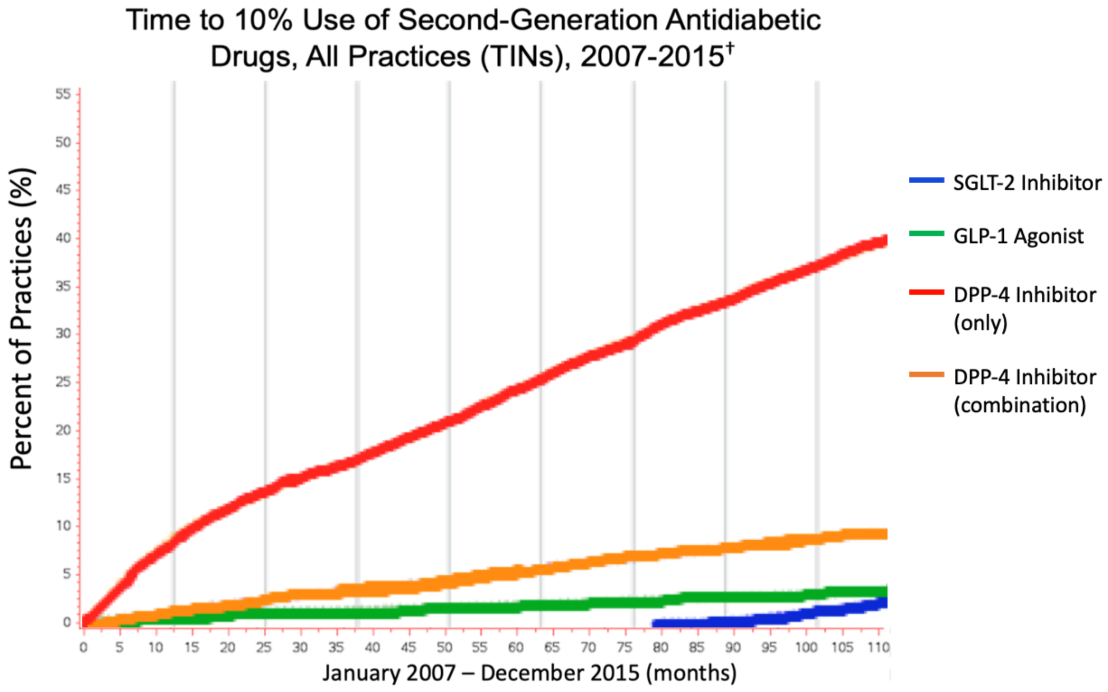

© 2020 Gilstrap LG et al. *JAMA Network Open.*

eTable 3. Comparison of Patients Dropped Owing to Small Practice Size or Inability to Attribute to a Practice vs Those Included in Initiation Cohort

**Binary Characteristics**						
	**Total N**	**Overall %**	**Patients in final cohort**			
			**No (excluded)**		**Yes (included)**	
			**N**	**%**	**N**	**%**
**All**	1655427	100	472339	100	1182233	100
**Year of Study**						
**2007**	206276	12.4606	62161	13.1603	143984	12.18
**2008**	182972	11.0529	58967	12.484	123855	10.48
**2009**	172258	10.4057	55773	11.8078	116414	9.85
**2010**	169379	10.2317	53040	11.2292	116270	9.83
**2011**	169677	10.2497	51557	10.9153	118053	9.99
**2012**	168210	10.1611	49364	10.451	118778	10.05
**2013**	176673	10.6724	48142	10.1923	128472	10.87
**2014**	201330	12.1618	47756	10.1105	153467	12.98
**2015**	208652	12.6041	45579	9.6496	162940	13.78
**Female**	951839	57.4981	279535	59.181	671831	56.83
**Male**	703588	42.5019	192804	40.819	510402	43.17
**Race**						
**White**	1232440	74.4485	317612	67.2424	914374	77.34
**Black**	168641	10.1872	63176	13.3751	105319	8.91
**Hispanic**	154784	9.3501	59674	12.6337	94937	8.03
**Other**	99562	6.0143	31877	6.7488	67603	5.72
**Population Density**						
**Urban**	1346357	81.3299	383774	81.2497	962472	81.41
**Large Rural**	148405	8.9648	35327	7.4792	113075	9.56
**Small Rural**	88037	5.3181	27456	5.8128	60578	5.12
**Isolated**	68996	4.1679	22887	4.8455	46108	3.90
**Geography**						
**Northeast**	282362	17.0567	93879	19.8753	188450	15.94
**Midwest**	388773	23.4848	95136	20.1415	293551	24.83
**South**	691515	41.7726	180027	38.1139	511327	43.25
**West**	286807	17.3253	97715	20.6875	188905	15.98
**Socioeconomics (ZCTA-level)**						
**Dual Eligibility**	444098	26.80%	185379	39.20%	258524	21.90%
**Disability**	227844	13.80%	72320	15.30%	155403	13.10%
**% Poverty**	238532	14.40%	80933	17.10%	157401	13.30%
**Comorbidities**						
**Acute Myocardial Infarction**	106009	6.40%	30255	6.41%	75706	6.40%

© 2020 Gilstrap LG et al. *JAMA Network Open.*

**Atrial Fibrillation**	280085	16.90%	79386	16.80%	200577	17.00%
**Chronic Kidney Disease**	455431	27.50%	138807	29.40%	316392	26.80%
**Congestive Heart Failure**	592467	35.80%	190624	40.40%	401562	34.00%
**Hyperlipidemia**	1416941	85.60%	385172	81.50%	1031039	87.20%
**Hypertension**	1521818	91.90%	432991	91.70%	1088044	92.00%
**Ischemic Heart Disease**	956726	57.80%	278924	59.10%	677313	57.30%
**Stroke/TIA**	305073	18.40%	104650	22.20%	200279	16.90%
**Cancer**	256431	15.50%	68440	14.50%	187840	15.90%
**Provider Characteristics**						
**Endocrinologist**	49821	3.01%	9580	2.03%	40207	3.40%
**Primary Care Physician**	1199441	72.50%	305119	64.60%	893735	75.60%
**Other Provider *** ***(has outpatient claims)***	208432	12.60%	68230	14.40%	140076	11.80%
**Hospital Based Provider** ***(no outpatient claims)***	197733	11.90%	89410	18.90%	108215	9.15%
**Provider Panel <250**	701350	42.40%	280182	59.30%	420779	35.60%
**Provider panel 250-500**	589361	35.60%	136726	28.90%	452351	38.30%
**Provider panel >500**	364716	22.00%	55431	11.70%	309103	26.10%
**Patients with diabetes in panel <25%**	265841	16.10%	90806	19.20%	174880	14.80%
**Patients with diabetes in panel 25-50%**	1063971	64.30%	272963	57.80%	790518	66.90%
**Patients with diabetes in panel >50%**	325615	19.70%	108570	23.00%	216835	18.30%
**Practice Characteristics**						
**Hospital Owned**	106009	6.40%	30255	6.41%	87721	7.42%
**Academic Center Affiliated**	280085	16.90%	79386	16.80%	56018	4.74%

**Continuous Characteristics**									
	**Overall Mean**	**Overall SD**	**Overall Range**	**Patients in final cohort**					
				**No (excluded)**			**Yes (included)**		
				**Mean**	**SD**	**Range**	**Mean**	**SD**	**Range**
**Age**	75.76	7.02	44	76.57	7.58	44	75.44	6.75	42
**Percent Poverty**	11.88	8.36	100	12.60	8.92	100	11.59	8.10	100
**Percent with High School Degree**	84.54	9.81	100	83.64	10.33	100	84.90	9.56	100
**Median Income ($)**	53324	21555	242089	53168	22536	242089	53386	21151	242089
**Hierarchical Condition Category Risk Score†**	2.31	2.00	19	2.62	2.29	18	2.19	1.85	19
**Number of Patients per Practice**	5065.54	9329.41	68400	212.88	189.19	4698	6512.51	10191.40	68393

© 2020 Gilstrap LG et al. *JAMA Network Open.*

**Number of Providers per Practice**	122.75	417.62	14221	7.26	49.35	4275	157.19	469.60	14221
**Number of Providers per Patient**	166.35	164.55	3228	134.78	125.67	1417	175.76	173.36	3228
**Provider Panel Size**	362.06	262.04	4815	272.84	224.03	4217	396.11	267.43	4815
**Percent of Provider's Panel with Diabetes**	0.41	0.16	1	0.43	0.17	1	0.40	0.16	1

* Prescriber has no face-to-face office visits in the outpatient or carrier file
[†] HCC scores of less than one are considered relatively healthy, higher numbers suggest of more comorbidities and disease complexity

HCC is hierarchical condition category; SD is standard deviation; TIA is transient ischemic attack

© 2020 Gilstrap LG et al. *JAMA Network Open.*

eTable 4. Comparison of Patients Dropped Owing to Missing Zip Code vs Those Included in Initiation Cohort

Binary Characteristics						
	Total N	Overall %	Patients in final cohort			
			No (excluded)		Yes (included)	
			N	%	N	%
**All**	1183088	100	855	100	1182233	100
**Year of Study**						
**2007**	144115	12.18	131	15.32	143984	12.18
**2008**	124005	10.48	150	17.54	123855	10.48
**2009**	116485	9.85	71	8.30	116414	9.85
**2010**	116339	9.83	69	8.07	116270	9.83
**2011**	118120	9.98	67	7.84	118053	9.99
**2012**	118846	10.05	68	7.95	118778	10.05
**2013**	128531	10.86	59	6.90	128472	10.87
**2014**	153574	12.98	107	12.51	153467	12.98
**2015**	163073	13.78	133	15.56	162940	13.78
**Female**	672304	56.83	473	55.32	671831	56.83
**Male**	510784	43.17	382	44.68	510402	43.17
**Race**						
**White**	914828	77.33	454	53.10	914374	77.34
**Black**	105465	8.91	146	17.08	105319	8.91
**Hispanic**	95110	8.04	173	20.23	94937	8.03
**Other**	67685	5.72	82	9.59	67603	5.72
**Population Density**						
**Urban**	962583	81.36	****	****	962472	81.41
**Large Rural**	113078	9.56	****	****	113075	9.56
**Small Rural**	60581	5.12	****	****	60578	5.12
**Isolated**	46109	3.90	****	****	46108	3.90
**Geography**						
**Northeast**	188483	15.93	****	****	188450	15.94
**Midwest**	293637	24.82	****	****	293551	24.83
**South**	511488	43.23	****	****	511327	43.25
**West**	189092	15.98	****	****	188905	15.98
**Socioeconomics (ZCTA-level)**						
**Dual Eligibility**	258719	21.90%	195	22.80%	258524	21.90%
**Disability**	155524	13.10%	121	14.20%	155403	13.10%
**% Poverty**	157599	13.30%	198	23.20%	157401	13.30%
**Comorbidities**						
**Acute Myocardial Infarction**	75754	6.40%	48	5.61%	75706	6.40%

© 2020 Gilstrap LG et al. *JAMA Network Open.*

**Atrial Fibrillation**	200699	17.00%	122	14.30%	200577	17.00%
**Chronic Kidney Disease**	316624	26.80%	232	27.10%	316392	26.80%
**Congestive Heart Failure**	401843	34.00%	281	32.90%	401562	34.00%
**Hyperlipidemia**	1031769	87.20%	730	85.40%	1031039	87.20%
**Hypertension**	1088827	92.00%	783	91.60%	1088044	92.00%
**Ischemic Heart Disease**	677802	57.30%	489	57.20%	677313	57.30%
**Stroke/TIA**	200423	16.90%	144	16.80%	200279	16.90%
**Cancer**	187991	15.90%	151	17.70%	187840	15.90%
**Provider Characteristics**						
**Endocrinologist**	40241	3.40%	34	3.98%	40207	3.40%
**Primary Care Physician**	894322	75.60%	587	68.70%	893735	75.60%
**Other Provider *** ***(has outpatient claims)***	140202	11.90%	126	14.70%	140076	11.80%
**Hospital Based Provider** ***(no outpatient claims)***	108323	9.16%	108	12.60%	108215	9.15%
**Provider Panel <250**	421168	35.60%	389	45.50%	420779	35.60%
**Provider panel 250-500**	452635	38.30%	284	33.20%	452351	38.30%
**Provider panel >500**	309285	26.10%	182	21.30%	309103	26.10%
**Patients with diabetes in panel <25%**	175035	14.80%	155	18.10%	174880	14.80%
**Patients with diabetes in panel 25-50%**	791008	66.90%	490	57.30%	790518	66.90%
**Patients with diabetes in panel >50%**	217045	18.30%	210	24.60%	216835	18.30%
**Practice Characteristics**						
**Hospital Owned**	87823	7.42%	102	11.90%	87721	7.42%
**Academic Center Affiliated**	56087	4.74%	69	8.07%	56018	4.74%

**Continuous Characteristics**									
	**Overall Mean**	**Overall SD**	**Overal l Range**	**Patients in final cohort**					
				**No (excluded)**			**Yes (included)**		
				**Mean**	**SD**	**Range**	**Mean**	**SD**	**Range**
**Age**	75.76	7.02	44	76.57	7.58	44	75.44	6.75	42
**Percent Poverty**	11.88	8.36	100	12.60	8.92	100	11.59	8.10	100
**Percent with High School Degree**	84.54	9.81	100	83.64	10.33	100	84.90	9.56	100
**Median Income ($)**	53324	21555	242089	53168	22536	242089	53386	21151	242089
**Hierarchical Condition Category Risk Score†**	2.31	2.00	19	2.62	2.29	18	2.19	1.85	19
**Number of Patients**	5065.54	9329.41	68400	212.88	189.19	4698	6512.51	10191.40	68393

© 2020 Gilstrap LG et al. *JAMA Network Open.*

**per Practice**									
**Number of Providers per Practice**	122.75	417.62	14221	7.26	49.35	4275	157.19	469.60	14221
**Number of Providers per Patient**	166.35	164.55	3228	134.78	125.67	1417	175.76	173.36	3228
**Provider Panel Size**	362.06	262.04	4815	272.84	224.03	4217	396.11	267.43	4815
**Percent of Provider's Panel with Diabetes**	0.41	0.16	1	0.43	0.17	1	0.40	0.16	1

* Prescriber has no face-to-face office visits in the outpatient or carrier file
† HCC scores of less than one are considered relatively healthy, higher numbers suggest of more comorbidities and disease complexity

HCC is hierarchical condition category; SD is standard deviation; TIA is transient ischemic attack

© 2020 Gilstrap LG et al. *JAMA Network Open.*
